# Two high-mobility group box domains act together to underwind and kink DNA

**DOI:** 10.1107/S1399004715007452

**Published:** 2015-06-30

**Authors:** R. Sánchez-Giraldo, F. J. Acosta-Reyes, C. S. Malarkey, N. Saperas, M. E. A. Churchill, J. L. Campos

**Affiliations:** a Departament d’Enginyeria Quimica, Universitat Politecnica de Catalunya, 08028 Barcelona, Spain; b Department of Pharmacology and the Program in Structural Biology and Biochemistry, University of Colorado School of Medicine, Aurora, CO 80045, USA

**Keywords:** high-mobility group protein, X-ray crystallography, DNA binding, HMGB1

## Abstract

High-mobility group protein 1 (HMGB1) is an essential and ubiquitous DNA architectural factor that influences a myriad of cellular processes. HMGB1 contains two DNA-binding domains, box A and box B, which have little sequence specificity but have remarkable abilities to underwind and bend DNA. Although HMGB1 box A is thought to be responsible for the majority of HMGB1–DNA interactions with pre-bent or kinked DNA, little is known about how it recognizes unmodified DNA. Here, the crystal structure of HMGB1 box A bound to an AT-rich DNA fragment is reported at a resolution of 2 Å. Two box A domains of HMGB1 collaborate in an unusual configuration in which the Phe37 residues of both domains stack together and intercalate the same CG base pair, generating highly kinked DNA. This represents a novel mode of DNA recognition for HMGB proteins and reveals a mechanism by which structure-specific HMG boxes kink linear DNA.

## Abstract 2

The crystal structure of HMGB1 box A bound to an unmodified AT-rich DNA fragment is reported at a resolution of 2 Å. A new mode of DNA recognition for HMG box proteins is found in which two box A domains bind in an unusual configuration generating a highly kinked DNA structure.

## 1. Introduction   

High-mobility group protein 1 (HMGB1) is a DNA architectural factor that affects numerous cellular processes by modulating chromatin structure (Thomas & Travers, 2001 ▸). It participates in the regulation of transcription, chromatin remodeling, recombination and DNA repair, and is requisite for transposition in gene therapy (Ivics *et al.*, 2004 ▸; Malarkey & Churchill, 2012 ▸; Štros, 2010 ▸). In an extracellular role, HMGB1 is a danger signal in inflammatory conditions, including autoimmunity and cancer (Klune *et al.*, 2008 ▸; Kang *et al.*, 2014 ▸; Yang *et al.*, 2013 ▸).

HMGB1 is the archetypal member of the HMGB proteins, a large family of proteins that includes many transcription factors and chromosomal proteins such as mammalian HMGB1–4, TFAM (mitochondrial transcription factor A), NHP6A/B (*Saccharomyces cerevisiae*) and HMGD (*Drosophila melanogaster*), as well as sequence-specific transcription factors such as TCF/LEF-1 and sex-determining factor SRY and SOX proteins among others (Malarkey & Churchill, 2012 ▸; Štros, 2010 ▸). The HMG box is the defining and characteristic domain of the HMGB family (Landsman & Bustin, 1993 ▸). This domain comprises three α-helices with an L-shaped structure, in which helix I and II form a short arm and helix III together with an N-terminal stretch of amino acids forms a long arm (Weir *et al.*, 1993 ▸; Read *et al.*, 1993 ▸; Jones *et al.*, 1994 ▸). Although many HMGB family members have only one HMG box, HMGB1 has two HMG boxes (A and B), the solution structures of which have been determined by NMR (Hardman *et al.*, 1995 ▸; Wang *et al.*, 2013 ▸; Weir *et al.*, 1993 ▸), and the HMG boxes are followed by an intrinsically disordered C-terminal tail.

**Figure 1:**
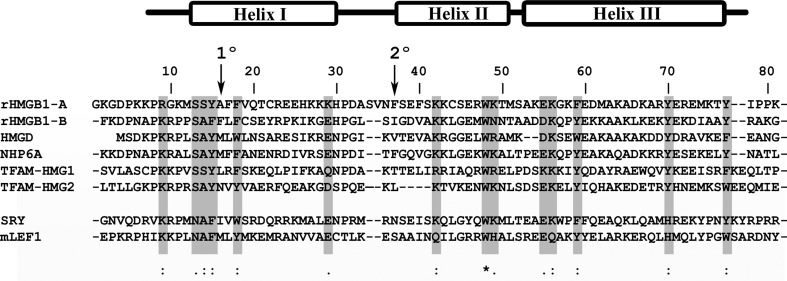
HMG-box sequence comparison. Sequence alignment of non-sequence-specific HMG-box proteins: HMGB1 (rat; rHMGB1-A, box A; rHMGB1-B, box B), HMGD (*Drosophila*), NHP6A (*S. cerevisiae*), sequence-specific/non-sequence-specific TFAM (human mitochondria; TFAM-HMG1, box A; TFAM-HMG2, box B) and sequence-specific SRY (human) and LEF1 (mouse). The three α-helices of the HMG box are shown above the alignment. Arrows indicate the 1° and 2° intercalating residues. Conserved residues (*Clustal Omega* alignment) are highlighted in gray, where an asterisk (*) indicates complete conservation, a colon (:) indicates conservation between groups with strongly similar properties and a dot (.) indicates conservation between groups with weakly similar properties.

A hallmark of HMGB proteins is their ability to recognize the minor groove of pre-bent, distorted or linear DNA, bending linear DNA between 70 and 180° towards the major groove (Dragan *et al.*, 2003 ▸, 2004 ▸; Werner *et al.*, 1995 ▸). The HMGB1 domains are unequal in these properties: box A recognizes both pre-bent (Teo, Grasser & Thomas, 1995 ▸) and linear DNA more tightly than box B (Müller *et al.*, 2001 ▸), but box B binds to mini-circles (Webb *et al.*, 2001 ▸) and bends linear DNA to a greater extent than box A (Paull *et al.*, 1993 ▸; Teo, Grasser & Thomas, 1995 ▸). This dramatic distortion of DNA is dependent on both shape complementarity and DNA intercalation of two apolar residues (Churchill *et al.*, 2010 ▸; Klass *et al.*, 2003 ▸; Murphy & Churchill, 2000 ▸; Roemer *et al.*, 2008 ▸). The primary intercalation residue is in helix I (1° in Fig. 1 ▸) and the second intercalation wedge (2° in Fig. 1 ▸) is at the start of helix II. HMGB1 box A is an exception because Ala16 at the 1° site cannot intercalate DNA but Phe37 at the 2° site can, and this is thought to be responsible for the superior ability of box A to recognize pre-bent DNA (reviewed by Štros, 2010 ▸). Indeed, the crystal structure of box A bound to cisplatin intrastrand GG cross-linked DNA showed Phe37 lodged into the cisplatin-induced kink, although the DNA itself was relatively undistorted compared with the free cisplatin-modified DNA (Ohndorf *et al.*, 1999 ▸). However, how box A recognizes natural, unmodified DNA remains unknown.

In order to understand how a structure-specific HMG box can recognize linear unmodified DNA, we determined the crystal structure of HMGB1 box A in complex with an AT-rich DNA fragment. Rigorous structural analysis of this structure revealed remarkable differences in the mode of DNA binding in comparison to non-sequence-specific HMG boxes bound to linear DNA (Murphy *et al.*, 1999 ▸; Murphy & Churchill, 2000 ▸; Allain *et al.*, 1999 ▸; Churchill *et al.*, 2010 ▸) and interesting similarities to the mode of binding observed for the pre-bent DNA (Ohndorf *et al.*, 1999 ▸).

## 2. Materials and methods   

### 2.1. Expression and purification of the protein and DNA   

Plasmid pGEX2T containing GST-tagged rat HMGB1 box A domain (Lys7–Pro80 in Fig. 1 ▸; Roemer *et al.*, 2008 ▸) was expressed in *Escherichia coli* Rosetta(DE3)pLysS strain. Cells were grown in the presence of ampicillin and chloramphenicol at 37°C with vigorous shaking until the absorbance at 600 nm reached 0.8. Expression of the fusion protein was then induced by the addition of 0.5 m*M* IPTG. The bacterial cells were harvested by centrifugation at 5000*g* for 10 min.

The protein was purified as follows. The resuspended pellet was sonicated in buffer 1 (20 m*M* Tris pH 7.9, 0.5 *M* NaCl, 10 m*M* EDTA, 1 m*M* DTT, 10% glycerol) with DNaseI and protease inhibitors (cOmplete protease-cocktail tablets, Roche). The clarified lysate was incubated with glutathione Sepharose 4B beads (GE Healthcare) pre-equilibrated with buffer 1 by rotating for 2 h at 4°C. The GST beads were then washed five times with wash buffer (20 m*M* Tris pH 7.9, 1 *M* NaCl, 10 m*M* EDTA, 1 m*M* DTT) and three times with thrombin buffer (20 m*M* Tris pH 7.9, 100 m*M* NaCl, 2.5 m*M* CaCl_2_, 1 m*M* DTT). The protein was cleaved with 200 U ml^−1^ thrombin by rotating overnight at 4°C and the protein was eluted from the beads with elution buffer (10 m*M* Tris pH 7.9, 100 m*M* NaCl, 10 m*M* EDTA, 1 m*M* DTT). The protein was further purified using a HiTrap SP FF cation-exchange column (GE Healthcare) pre-equilibrated with elution buffer. The protein was eluted with a linear gradient to a final concentration of 1 *M* NaCl in the same buffer. The most pure fractions as assessed by SDS–PAGE were pooled and concentrated for final purification *via* size-exclusion chromatography (Superdex 75 16/600, GE Healthcare). Pure fractions, based on analysis by SDS–PAGE, were pooled, dialyzed (25 m*M* HEPES pH 7.4 at 4°C, 75 m*M* NaCl, 1 m*M* DTT) and concentrated to 54 mg ml^−1^ by ultrafiltration (Vivaspin, GE Healthcare; Microcon, Millipore). The final protein concentration was calculated from the *A*_280_ using an extinction coefficient of 9770 *M* ^−1^ cm^−1^ calculated using *Peptide Property Calculator* v.1.0 (A. Chazan, Northwestern University, Illinois, USA; http://www.basic.northwestern.edu/biotools/proteincalc.html). The protein mass was confirmed by matrix-assisted laser desorption/ionization mass spectrometry (MALDI-TOF) by comparison of the experimental molecular-weight value (8924 Da) and the theoretical value (8929 Da).

The d(ATATCGATAT)_2_ oligonucleotide, synthesized in an automatic synthesizer by the phosphoramidite method and purified by gel filtration and reverse-phase HPLC, was supplied by the Pasteur Institute. The DNA was dissolved in 25 m*M* sodium cacodylate pH 6.5 buffer. The final concentration was calculated from the *A*_260_ using an extinction coefficient of 106.2 m*M* ^−1^ cm^−1^.

### 2.2. Electrophoretic mobility shift assays (EMSAs)   

DNA-binding assays were performed on nondenaturing 6% polyacrylamide gels. 1 µ*M* oligonucleotide and increasing concentrations of box A domain were incubated for 30 min in 0.33× TBE (30 m*M* Tris–borate, 0.66 m*M* EDTA) and 3% glycerol. Electrophoresis was carried out at 125 V for 30 min at 4°C. Gels were stained with SYBR Gold (Life Technologies) and visualized with UV light using a Gel Doc XR (Bio-Rad).

### 2.3. Supercoiling assays   

0.3 µg of relaxed pSTATCEN plasmid (∼4.5 kb) was prepared by treatment of the supercoiled DNA with topo­isomerase I at 37°C for 1 h. Extra topoisomerase I and increasing amounts of box A domain or didomain AB were added to the reactions. The reaction mixtures were incubated at 37°C for 1 h in two different buffers: 100 m*M* NaCl, 5 m*M* MgCl_2_, 35 m*M* Tris pH 7.5, 1 m*M* DTT (high ionic strength) or 10 m*M* NaCl, 5 m*M* MgCl_2_, 35 m*M* Tris pH 7.5, 1 m*M* DTT (low ionic strength). Reactions were stopped by the addition of 0.5% SDS and 0.25 mg ml^−1^ proteinase K and incubation at 37°C for 30 min. Electrophoresis of topoisomer populations was carried out in 1% agarose gel in 1× TPE (90 m*M* Tris–phosphate, 2 m*M* EDTA) at 90 V for 2 h. The gels were stained with SYBR Gold (Invitrogen, Life Technologies) and photographed with UV transillumination.

### 2.4. Crystallization, data collection and structure determination   

Crystals of HMGB1 box A bound to d(ATATCGATAT)_2_ were obtained by the hanging-drop vapor-diffusion method. The protein–DNA complex was obtained by incubation (with final concentrations of 1.6 m*M* protein and 0.8 m*M* DNA) for approximately 1 h at 4°C. A hanging drop consisting of 1.5 µl complex solution and 1.5 µl buffer from the Natrix screen (Hampton Research) consisting of 40 m*M* MgCl_2_, 50 m*M* sodium cacodylate pH 6.0, 5% 2-methyl-2,4-pentanediol (MPD) was equilibrated against 40% MPD. High-quality needle-shaped crystals obtained from this drop (∼10 × 150 µm) were flash-cooled and stored in liquid nitrogen. X-ray data were collected on the BL13-XALOC beamline at the ALBA synchrotron, Barcelona, Spain (λ = 0.97949 Å) using a PILATUS 6M detector (Dectris).

The data were processed with *HKL*-2000 (Otwinowski & Minor, 1997 ▸). The space group of the complex was *P*2_1_2_1_2_1_, with unit-cell parameters *a* = 42.79, *b* = 84.29, *c* = 94.31 Å, as confirmed with *POINTLESS* (Evans, 2006 ▸). Assuming the presence of two DNA duplexes and two protein molecules in the asymmetric unit, the Matthews coefficient was estimated to be 2.82 Å^3^ Da^−1^, with a solvent content of ∼60% (Kantardjieff & Rupp, 2003 ▸; Matthews, 1968 ▸).

In a first unsuccessful attempt to solve the structure, an ideal B-DNA was constructed with *TURBO-FRODO* (Roussel *et al.*, 1998 ▸). This DNA and the full protein coordinates of HMGB1 box A (Pro8–Tyr77 in Fig. 1 ▸; Ohndorf *et al.*, 1999 ▸; PDB entry 1ckt) were used as a search model for molecular replacement. Finally, the structure was solved by trimming the DNA model and using *Phaser* (McCoy *et al.*, 2005 ▸). Two d(ATAT)_2_ fragments were located and placed at the appropriate angle as indicated by the orientation of the stacking reflections. Next, the two HMGB1 box A models were added, one by one, to the structure using *MOLREP* (Vagin & Teplyakov, 2010 ▸) and fitted in accordance with the previously placed DNA fragments. The missing central CG base pairs of the duplex and the missing protein residues were added using *Coot* (Emsley & Cowtan, 2004 ▸). Finally, a second straight DNA duplex was located with *MOLREP*. Real-space refinement was performed with *Coot*. At this point, we were surprised to find that the asymmetric unit contained two different DNA duplexes: one bent and complexed with two proteins and the other free and straight (Supplementary Fig. S3). We carried out maximum-likelihood refinement using *REFMAC*5 (Murshudov *et al.*, 2011 ▸). After several cycles, noncrystallographic symmetry restraints were applied, and TLS refinement was performed in the last round. The structure was validated with *Coot* and *MolProbity* (Chen *et al.*, 2010 ▸). Electron density for the C-terminal Pro80 in both box A domains was not observed. The average root-mean-square deviation (r.m.s.d.) between the C^α^ atoms of the two box A domains was 0.49 Å, with an r.m.s.d. of 0.94 Å for all atoms. Details of data and refinement statistics are given in Table 1 ▸.

DNA parameters were calculated using 3*DNA* (Lu & Olson, 2003 ▸). The axis of the oligonucleotide was obtained with *Curves*+ (Lavery *et al.*, 2009 ▸). A schematic diagram of the protein–nucleic acid interactions was drawn using *NUCPLOT* (Luscombe *et al.*, 1997 ▸). Figures were prepared with *PyMOL* (Schrödinger) and *Coot* (Emsley & Cowtan, 2004 ▸). The r.s.m.d. values and the superimposed models for the different HMG box A domains were obtained using *SUPERPOSE* (Sievers *et al.*, 2011 ▸). Amino-acid sequences were aligned using *Clustal Omega* (Maiti *et al.*, 2004 ▸) with UniProt accession numbers P63159 (HMGB1), Q05783 (HMGD), P11632 (NHP6A), Q00059 (TFAM), Q05066 (SRY) and P27782 (mLEF1) (The UniProt Consortium, 2014 ▸).

## 3. Results   

### 3.1. General view of the structure   

We have determined the crystal structure of HMGB1 box A bound to the linear duplex DNA d(ATATCGATAT)_2_ (Table 1 ▸). The interaction between box A and DNA was verified by electrophoretic mobility shift assays (Supplementary Fig. S1*a*). The refined model at a resolution of 2.0 Å was well resolved (Supplementary Fig. S2*a*), with an asymmetric unit comprising one unbound straight DNA duplex and one bent DNA duplex bound by two box A domains (Supplementary Fig. S3*a*). These duplexes form a pseudo-continuous helix throughout the crystal (Supplementary Fig. S3*b*). The structure also contains a single hexahydrated magnesium ion and a network of water molecules (Supplementary Fig. S2*b*).

**Figure 2:**
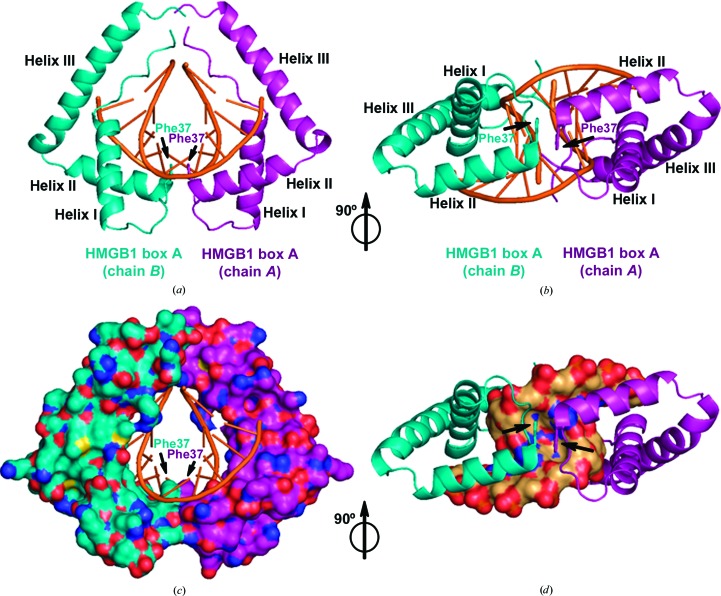
The two near-symmetric box A domains collaborate to bend DNA. (*a*) View of both domains enclosing the kinked DNA. The HMGB1 box A domains are colored purple and cyan, whereas the kinked DNA is colored orange. Phe37 of both domains is indicated. (*b*) View showing the 2° intercalation site, with the two phenylalanines at the central CG base pair. (*c*) Surface representation of the two box A domains. (*d*) Surface representation of the DNA showing the pocket enclosing the two Phe37 residues (indicated by arrows).

The two box A domains bind in an approximately symmetric manner about the dyad axis of the palindromic DNA decamer, with water-mediated interactions between the domains (Supplementary Fig. S4). Molecule *A* contacts one half of the duplex, from A_1_/T_20_ to C_5_/G_16_, and molecule *B* contacts the other half, from G_6_/C_15_ to T_10_/A_11_ (Figs. 2 ▸ *a*, 2 ▸ *b* and 3 ▸ *a*). The two domains enclose the DNA (Figs. 2 ▸ *a* and 2 ▸ *c*), unwinding and bending it by approximately 85°, with intercalation of the two Phe37 residues at the central CG base pair (Figs. 2 ▸ *b* and 2 ▸ *d* and Supplementary Table S1). This tail-to-tail mode of binding places both Phe37 side chains in a cleft created in the DNA minor groove (Figs. 2 ▸ *b* and 2 ▸ *d*), producing a prominent kink in the DNA towards the major groove. The two phenyl rings of Phe37 are parallel to each other at 3.5 Å, a distance indicative of π-stacking. These features contrast with the other multi-domain HMG-box–DNA structures: HMGD domains interact in a head-to-head orientation (Murphy *et al.*, 1999 ▸), SRY.B domains bind in a head-to-head fashion with the two 2° intercalation sites separated by 16 bp (Stott *et al.*, 2006 ▸) and TFAM HMG domains bind tail to tail but the two 2° intercalation sites are separated by 11 bp (Ngo *et al.*, 2011 ▸, 2014 ▸; Rubio-Cosials *et al.*, 2011 ▸). Thus, this collaborative binding mode, whereby the 2° intercalation residues of two HMG box A domains act in the same base step, has not previously been observed.

### 3.2. Similarities to other HMG boxes   

**Figure 3:**
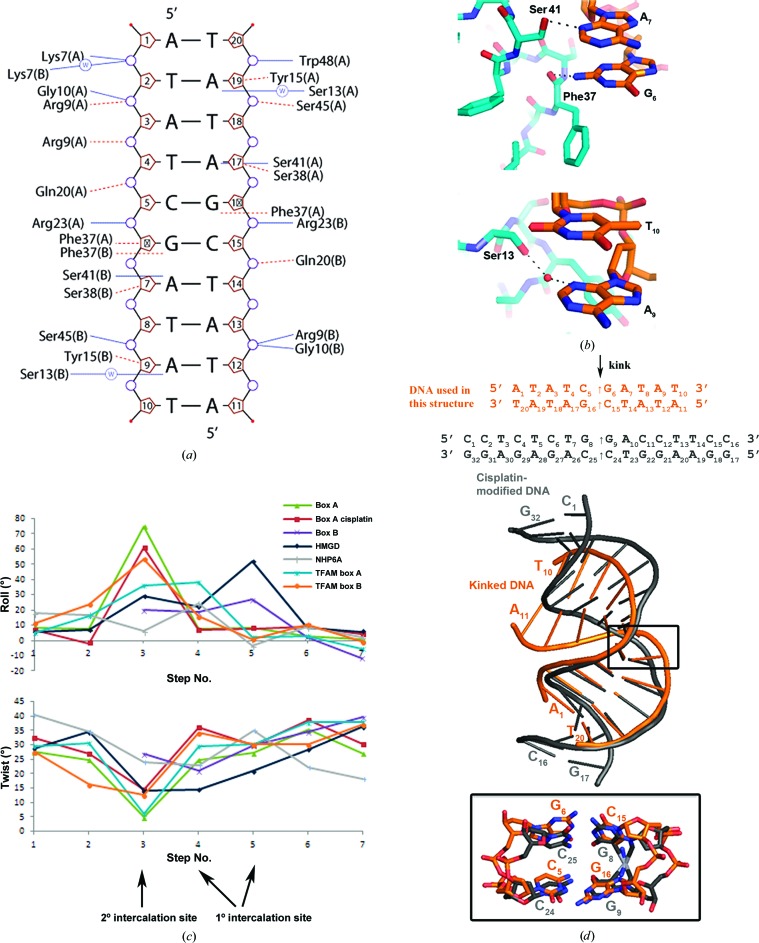
The kinked DNA structure. (*a*) DNA–box A contacts (*NUCPLOT*). Hydrogen bonds are shown as solid lines and nonbound contacts are shown as dashed lines. (*b*) Close-up views of the protein–DNA purine base interactions. Hydrogen bonds from residues Phe37 and Ser41 to base pairs A_7_ and G_6_ and a water-mediated hydrogen bond from Ser13 to A_9_ are shown in the upper and lower diagrams, respectively. (*c*) Comparison of DNA parameters for HMG-box intercalation sites. The roll and twist angles for box A in this structure were obtained with 3*DNA*, and those for PDB entries 1ckt (box A, cisplatin; Ohndorf *et al.*, 1999 ▸), 2gzk (box B; Stott *et al.*, 2006 ▸), 1qrv (HMGD; Murphy *et al.*, 1999 ▸), 1j5n (NHP6A; Masse *et al.*, 2002 ▸) and 3tmm (TFAM; Ngo *et al.*, 2011 ▸) were taken from the Nucleic Acids Data Bank (NDB; see also Supplementary Table S2). (*d*) Superimposition of box A kinked DNA (orange) with cisplatin-modified DNA (grey).

The interactions of both box A molecules with DNA (Fig. 3 ▸ *a*) share many features with other HMGB–DNA complexes. The overall orientations with respect to the DNA of the N-terminal stretch and globular core are conserved (Figs. 2 ▸ *c* and 2 ▸ *d*). Hydrogen bonds from Arg23 and Trp48 to the sugar-phosphate backbone are also well conserved; specifically, this was observed in DNA complexes with SRY.B (Stott *et al.*, 2006 ▸), DNA–cisplatin–box A (Ohndorf *et al.*, 1999 ▸), HMGD (Murphy *et al.*, 1999 ▸), NHP6A (Allain *et al.*, 1999 ▸), SRY (Werner *et al.*, 1995 ▸) and LEF1 (Love *et al.*, 1995 ▸). In fact, Trp48 has been found to be important for the supercoiling activity of HMGB1 box A (Teo, Grasser, Hardman *et al.*, 1995 ▸). Despite this overall conservation of protein–DNA interactions, the HMG boxes HMGB1 box B, HMGD, TFAM and NHP6A differ in their DNA-bending properties. They bend DNA over more than one base-pair step (Fig. 3 ▸ *c*) rather than at just a single base step as observed here for box A, which gives rise to the DNA kink.

### 3.3. Unique features of the complex   

The interaction of Phe37 with the DNA kink is central to the unique mode of DNA recognition seen in this HMGB box A–DNA structure. Phe37 is also important for the recognition of structured DNA, as He *et al.* (2000 ▸) discovered when the Phe37Ala mutant no longer bound to pre-bent DNA. In our structure, Phe37 forms hydrogen bonds to G_6_ (N2) (Fig. 3 ▸ *b*) and is buttressed by van der Waals contacts between Ser38 and the deoxyriboses of G_6_ and A_7_ adjacent to Phe37. The hydroxyl H atom of Ser41 forms a hydrogen bond to A_7_ (N3) and van der Waals contacts with G_6_ and A_7_. However, this interaction of Phe37 and Ser41 with GA base pairs was also found in the structure of cisplatin–DNA–box A.

A feature of HMGB1 is its ability to bind to DNA in a non-sequence-specific fashion. It is thought that the two equivalent residues in LEF1 (Ser37 and Asn41) and SRY (Ser38 and Ser41) contribute to their sequence specificity because these residues form direct hydrogen bonds to the DNA bases (Werner *et al.*, 1995 ▸; Love *et al.*, 1995 ▸). Residue 13, which has also been implicated in the sequence specificity of these transcription factors, is here a serine that makes a water-mediated hydrogen bond to A_9_ (N3) (Fig. 3 ▸ *b*). Interestingly, this interaction is not observed in the cisplatin–DNA–box A complex. However, equivalent interactions of this serine with DNA in the HMGD (Murphy *et al.*, 1999 ▸) and HMGB1 box B (Stott *et al.*, 2006 ▸) structures have been observed, but they did not contribute to the sequence specificity of the HMG box (Klass *et al.*, 2003 ▸). Therefore, although in the HMG box A–DNA structure Ser13 together with Ser41 and Tyr15 participates in a water network that interconnects the central bases A_3_, T_4_ and C_5_ (and A_13_, T_14_ and C_15_), this is not expected to contribute to any sequence selectivity of box A.

### 3.4. DNA deformation   

The distortion of the DNA induced by box A domains is remarkably similar to that imposed solely by the cisplatin cross-link. At the kink in the box A–DNA structure, the minor groove widens, the major groove narrows and the DNA is underwound (Supplementary Fig. S1*b*, Table S1 and Movie S1); in particular, the C_5_G_6_/C_15_G_16_ base step has a roll angle of 74.85°, a twist angle of 4.82° and a rise of 6.64 Å, compared with standard values of a roll of 0.60°, a twist of 36.00° and a rise of 3.32 Å for B-DNA (Olson *et al.*, 2001 ▸). These DNA deformations are only slightly larger than those seen in the cisplatin-modified DNA–box A structure (Ohndorf *et al.*, 1999 ▸), where the roll values at the kink are 74.85 and 60.61°, respectively (Figs. 3 ▸ *c* and 3 ▸ *d*). The r.m.s.d. between the DNA duplex of this structure and the box A–cisplatin-modified DNA (Ohndorf *et al.*, 1999 ▸) is 3.23 Å, and is 2.59 Å for a similar, but unbound, cisplatin-modified DNA (Takahara *et al.*, 1996 ▸). Thus, the collaborative binding of both box A domains distorts DNA similarly to cisplatin alone.

### 3.5. Box A structure and comparisons   

**Figure 4:**
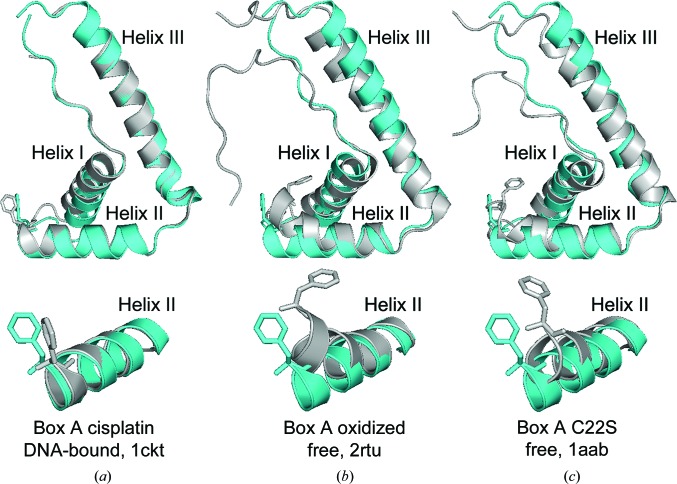
Comparison of box A structures and Phe conformations. Superimposition of box A from this structure (cyan) with (*a*) box A from cisplatin-modified DNA (PDB entry 1ckt; gray; Ohndorf *et al.*, 1999 ▸), (*b*) box A from the solution structure of the oxidized form (PDB entry 2rtu; gray; Wang *et al.*, 2013 ▸) and (*c*) the C22S mutant box A free reduced form (PDB entry 1aab; gray; Hardman *et al.*, 1995 ▸).

The structure of box A adapts to unmodified DNA differently than to cisplatin-modified DNA. The overall r.m.s.d. for the box A domains in the two structures is 1.68 Å (Fig. 4 ▸ *a* and Supplementary Table S2). The main differences are found near Phe37, in the loop between helix I and II, and in helix I, which is straighter when box A is bound to cisplatin-modified DNA. However, in both box A–DNA structures helix II is relatively straight, unlike any of the other free HMG box A structures (Fig. 4 ▸). This configuration of helix II might facilitate the interaction of Phe37 with the DNA kink site and shows that box A can adopt different conformations in different contexts.

The orientation of Phe37 is altered by the oxidation of Cys residues in HMGB1 (Wang *et al.*, 2013 ▸). One consequence of such oxidation is the shuttling of the oxidized HMGB1 out of the nucleus to the cytosol and extracellular matrix, where it can serve as a damage-associated molecular pattern (DAMP; Kang *et al.*, 2014 ▸; Sims *et al.*, 2010 ▸; Malarkey & Churchill, 2012 ▸). Therefore, understanding the mechanism by which the oxidized and reduced forms of HMGB1 lead to the observed decreased DNA-binding affinity is of particular biological interest. The solution structure of oxidized box A in an unbound state (Wang *et al.*, 2013 ▸) differs considerably from the box A–DNA structure (Fig. 4 ▸), with r.m.s.d. values of 3.46 and 2.73 Å overall and for α-helices only, respectively (Supplementary Table S2). In the oxidized form, helix II of box A is bent towards helix III and the phenyl ring of Phe37 is now further from the position needed to intercalate the DNA (Figs. 4 ▸ *b* and 4 ▸ *c*). Additionally, the loop between helices I and II is nearer helix I in the oxidized box A, and helices I and II are closer to each other owing to the disulfide bridge between Cys22 and Cys44. This comparison provides an explanation of how oxidation of box A can result in decreased DNA-binding affinity.

## 4. Discussion   

Previous structural studies have indicated that the box A domain binds to noncanonical DNA, for example four-way junctions (Webb & Thomas, 1999 ▸) and cisplatin-modified DNA (Ohndorf *et al.*, 1999 ▸). In contrast, our work not only demonstrates the ability of the HMGB1 box A domain to bind linear unmodified DNA, but also reveals a new mode of DNA recognition for HMG-box proteins, in which two domains act together to underwind and kink DNA. Thus, the HMGB1 box A–DNA structure reported here shows two important features: the changes that the box A domain causes in linear unmodified DNA and their ability to act in a concerted way.

HMGB1 is ubiquitously expressed at a very high level in the cell (an average of 10^6^ molecules; Catez *et al.*, 2004 ▸) and it is known that it is overexpressed in most tumors, including leukemia, hepatocellular carcinoma and gastric and colorectal adenocarcinomas (reviewed by Müller *et al.*, 2004 ▸). It is thus tempting to speculate that such situations might favor the formation of complexes in which two protein molecules are involved in DNA binding.

### 4.1. The HMGB1 box A domain distorts linear DNA   

The interaction of two box A domains creates the largest distortion of the roll and twist angles in a base-pair step observed to date for an HMG box (Stott *et al.*, 2006 ▸; Ohndorf *et al.*, 1999 ▸; Murphy *et al.*, 1999 ▸; Allain *et al.*, 1999 ▸; Ngo *et al.*, 2011 ▸, 2014 ▸; Rubio-Cosials *et al.*, 2011 ▸). Interestingly, the HMG boxes from HMGD and NHP6A, as well as sequence-specific HMG-box domains, are structurally more similar to box B than to box A (Stott *et al.*, 2006 ▸; Ohndorf *et al.*, 1999 ▸; Murphy *et al.*, 1999 ▸; Allain *et al.*, 1999 ▸). Thus, the structural differences of box A and box B might relate to their ability to distort DNA differently, for example one kinking and the other smoothly bending the DNA.

Despite the observations that the HMG boxes of HMGB1 do not show any sequence specificity (Teo, Grasser & Thomas, 1995 ▸), in the box A–DNA structure we find that the intercalation of Phe37 occurs in the pyrimidine–purine base step CG. The pyrimidine–purine steps are the most deformable sequence in DNA and show a high flexibility in many protein–DNA complexes (Olson *et al.*, 1998 ▸). It was also found to be a favored base step in binding-site selection studies of HMGD (Churchill *et al.*, 1995 ▸). Remarkably, a mutant of HMGD, HMGD-M13A, which loses the ability to intercalate DNA at the 1° site, has the 2° intercalating residue also located between a pyrimidine–purine step (Churchill *et al.*, 2010 ▸). These similarities in intercalation-site sequence support the model that structure-specific binding of HMGB proteins is based on the deformability of their binding substrates (Murphy & Churchill, 2000 ▸).

### 4.2. Oligomerization of HMG proteins in DNA binding   

A distinctive feature of our structure is the presence of two HMGB domains acting together on the same DNA-binding site. This is the first time that such a joint action has been reported.

Oligomerization of individual HMGB1 boxes and a HMGB didomain has been observed when bound to supercoiled circular and linear DNA, as reported by Teo, Grasser & Thomas (1995 ▸) in cross-linking assays. Additionally, HMGB1 exhibits cooperative binding to DNA mini-circles (Webb *et al.*, 2001 ▸). Finally, in electron-microscopy experiments, oligomeric protein ‘beads’ were observed at the bases of the loops and at the crossovers created by the didomain on circular and linear DNA, which could lead to DNA compaction (Štros, Štokrová *et al.*, 1994 ▸; Štros, Reich *et al.*, 1994 ▸).

Other observations of HMG-box associations include TFAM and HMGD. TFAM binds to the mitochondrial genomic DNA, compacting it into the mitochondrial nucleoid (Kaufman *et al.*, 2007 ▸). Interestingly, recent crystallographic studies of the structure of TFAM bound to DNA (Ngo *et al.*, 2011 ▸, 2014 ▸; Rubio-Cosials *et al.*, 2011 ▸) showed a crystal-packing contact mediated by the interaction of two HMG box A helices III (Ngo *et al.*, 2014 ▸). Substitution of amino-acid residues designed to disrupt this interaction led to a mutant of TFAM that had a decreased ability to compact DNA but that retained the ability to bind DNA, bend DNA and activate transcription (Ngo *et al.*, 2014 ▸). For the single HMG-box protein HMGD, cooperative binding to linear DNA giving rise to multimeric complexes has been observed (Churchill *et al.*, 1999 ▸). The crystal structures of both the HMG box of HMGD (Murphy *et al.*, 1999 ▸) and an HMGD intercalation mutant bound to DNA (Churchill *et al.*, 2010 ▸) showed interactions of helix III either from adjacent HMG boxes within the asymmetric unit or from HMG boxes at the sites of crystal-packing contacts. Moreover, HMGD exhibited head-to-head and head-to-tail binding orientations. Although it is not known which of these modes of oligomerization HMGD uses *in vivo*, the observation of similar types of HMG box–HMG box interactions in quite different HMGB proteins suggests that there are multiple ways in which HMG boxes can bind, bend and compact DNA.

**Figure 5:**
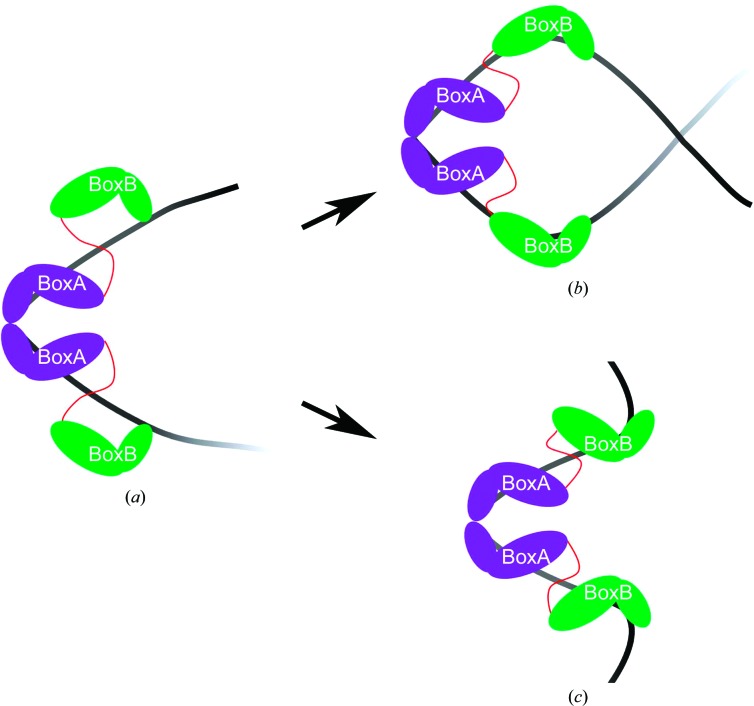
Schematic model of the organization of two HMGB1 molecules with each box A bound to the same DNA-binding site, as in our structure. The binding of box A (in purple) of both proteins through the Phe37 pair kinks the DNA by about 85° (*a*). The binding of each box B domain (in green) can originate the formation of a loop (*b*) or other DNA conformation (*c*). For simplicity, the acidic tails have not been drawn.

In our structure of HMGB1 box A, the two boxes could either come together to bind DNA or the binding of one box A could facilitate the binding of the second box. In Fig. 5 ▸ we show a model of how DNA could be bent when the binding of two whole HMGB1 proteins (with box A and box B) is considered. Besides the kinking of DNA imposed by the binding of the two boxes A (Fig. 5 ▸ *a*), the binding of the box B of both molecules could originate a loop (Fig. 5 ▸ *b*) or other conformations (Fig. 5 ▸ *c*) in DNA.

### 4.3. Chromatin modulation by HMGB1 and H1   

The binding of the box A domain to B-DNA is of utmost biological importance since HMGB1 is a key architectural protein in chromatin and subtle changes such as oxidation have dramatic functional consequences. It has been established that H1 and HMGB1 can contribute to modulation of the chromatin structure and both present similar binding sites within linker DNA (Štros, 2010 ▸). It has been repeatedly proposed that HMGB1 could displace linker H1 histones from DNA or chromatin (reviewed by Thomas & Stott, 2012 ▸; Ner *et al.*, 2001 ▸; Jackson *et al.*, 1979 ▸). Recent studies demonstrate that oxidized HMGB1 has a limited capacity for H1 displacement and the redox state of HMGB1 modulates the ability to bind and bend DNA (Polanska *et al.*, 2014 ▸). Our comparison of the structures of oxidized box A (with the disulfide bridge Cys22–Cys44) with box A bound to DNA in our structure provides an explanation for the decrease of affinity owing to the different availability of Phe37 to intercalate DNA.

In conclusion, we show how box A is able to bind linear unmodified DNA, unwind it and create a kink of 85° by means of two box A domains acting together in a symmetric manner. Our results open the possibility that the simultaneous binding of these two domains could be indicative of a concerted action of two HMGB1 molecules to bend DNA *in vivo*. Further research is required to ascertain whether this concerted binding is cooperative and whether it can also be extended to other HMG-box-containing proteins.

## Supplementary Material

PDB reference: HMGB box A bound to AT-rich DNA, 4qr9

Supplementary Tables and Figures.. DOI: 10.1107/S1399004715007452/mh5175sup1.pdf

Supplementary Movie S1. DNA distortion induced by two box A domains. The video presents the transition from B-DNA to the kinked DNA of this structure.. DOI: 10.1107/S1399004715007452/mh5175sup3.mp4

## Figures and Tables

**Table 1:** Data and refinement statistics Values in parentheses are for the highest resolution shell.

Data collection	
Space group	*P*2_1_2_1_2_1_
Unit-cell parameters (, )	*a* = 42.8, *b* = 84.2, *c* = 94.2, = = = 90.0
Resolution ()	42.122.00 (2.072.00)
*R*_merge_ (%)	11.3 (65.8)
*I*/(*I*)	16.0 (1.90)
Completeness (%)	98.9 (93.5)
Multiplicity	7.1 (5.1)
Refinement	
No. of reflections	22219
*R*_work_/*R*_free_ (%)	19.9/23.4
Wilson *B* factor (^2^)	35.7
No. of atoms	
Protein	1254
DNA	808
Mg^2+^	1
Water	115
Total	2178
*B* factors (^2^)	
Protein	44.4
DNA	37.6
Mg^2+^	21.1
Average	42.4
R.m.s. deviations	
Bond lengths ()	0.016
Bond angles ()	1.62
Ramachandran plot statistics (%)	
Most favored region	98.54
Allowed region	1.46
Disallowed region	0
PDB code	4qr9
