# Supplementary material for: Two high-mobility group box domains act together to underwind and kink DNA
Source: Acta Crystallogr D Biol Crystallogr. 2015 Jun 30;71(Pt 7):1423–32. doi: 10.1107/S1399004715007452 (PMC4498601; doi:10.1107/S1399004715007452)
Supplement: Supplementary file 1 [file d-71-01423-sup1.pdf]

BIOLOGICAL CRYSTALLOGRAPHY

Volume 71 (2015)

Supporting information for article:

Two high-mobility group box domains act together to underwind and kink DNA

R. Sánchez-Giraldo, F. J. Acosta-Reyes, C. S. Malarkey, N. Saperas, M. E. A. Churchill and J. L. Campos

Table S1 DNA structural parameters

Roll and twist angles for some of the HMG-box proteins. The symbol ↓ indicates the intercalating residues. The roll and twist profiles have been plotted aligning the HMG-box sequences in relation to the second site of intercalation (orange). The first site of intercalation is also highlighted (blue).

2º Intercalation site

1º Intercalation site

	Box A (4QR9) X-ray[1]				Box A cisplatin (1CKT) X-ray[2]				Box B (2GZK) NMR[2]			
	Step	↓	Roll (º)	Twist (º)	Step	↓	Roll (º)	Twist (º)	Step	↓	Roll (º)	Twist (º)
1	AT/AT		8.4	27.5	CT/AG		6.9	32.3	-	-	-	-
2	TG/CA		7.9	24.8	TG/CA		-1.9	27.0	-	-	-	-
3	CG/CG	Phe	74.8	4.9	GG/CC	Phe	60.6	14.4	GG/CC	Ile	19.8	27.0
4	GA/TC		8.0	24.8	GA/TC		6.8	36.1	GG/CC		18.5	21.1
5	AT/AT		8.4	27.3	AC/GT		7.9	29.7	GA/TC	Phe	26.7	29.7
6	TA/TA		2.5	35.2	CC/GG		9.3	38.6	AT/AT		2.3	34.5
7	AT/AT		0.6	27.0	CT/AG		4.0	30.4	TC/GA		-11.9	39.7

	HMGD (1QRV) X-ray[2]				NHP6A (1J5N) NMR[2,3]				TFAM box A (3TMM) X-ray[2]				TFAM box B (3TMM) X-ray[2,3]			
	Step	↓	Roll (º)	Twist (º)	Step	↓	Roll (º)	Twist (º)	Step	↓	Roll (º)	Twist (º)	Step	↓	Roll (º)	Twist (º)
1	GC/GC		5.2	28.8	GA/TC		17.8	40.3	AC/GT		5.2	29.4	CC/GG		11.0	27.5
2	CG/CG		6.7	34.5	AA/TT		16.9	34.7	CT/AG		16.1	30.6	CC/GG		23.5	16.3
3	GA/TC	Val, Thr	29.0	14.1	AC/GT	Phe	6.0	24.0	TG/CA	Ile	36.0	6.1	CA/TG	Pro, Leu	53.5	12.6
4	AT/AT		22.1	14.6	CA/TG	Tyr, Met	24.2	22.8	GT/AC	Leu	38.2	29.5	AA/TT	Asn	15.6	34.2
5	TA/TA	Met	51.6	21.0	AA/TT		-3.7	35.0	TT/AA		2.4	30.1	AC/GT		1.0	30.3
6	AT/AT		8.7	28.5	AT/AT		9.0	22.2	TA/TA		3.0	37.7	CT/AG		10.4	30.2
7	TC/GA		5.7	36.3	TC/GA		1.8	18.0	AA/TT		-5.3	38.0	TA/TA		-1.0	37.0

[1]Parameters calculated with 3DNA. [2]According to nucleic acids data bank (NDB) for 1CKT, 2GZK, 1QRV, 1J5N and 3TMM. [3]Sequences read in the opposite sense to coincide with the intercalation sites.

Table S2 R.m.s.d. table

R.m.s.d. values (in Å) for the comparison of the box A (chain B) of this structure with other HMGB1 box A proteins. These values were obtained with SuperPose V1.0 (Maiti *et al.*, 2004) and used the model 1 for the NMR structures. Note: Chain B was used as the reference for comparisons as it showed better B-factors than chain A.

	All protein atoms[1] (Å)			Alpha helix atoms[2] (Å)		
	Cα	Backbone	All	Cα	Backbone	All
**Chain A (from this structure)**	**0.46**	**0.46**	**0.95**	**0.34**	**0.33**	**1.01**
**Box A – 1CKT (X-ray Cisplatin-DNA)**	**1.23**	**1.20**	**1.68**	**1.05**	**1.06**	**1.61**
**Box A – 1AAB (NMR – reduced form)**	**2.55**	**2.63**	**3.08**	**1.72**	**1.75**	**2.50**
**Box A – 2RTU (NMR – oxidized form)**	**2.58**	**2.54**	**3.46**	**2.01**	**1.97**	**2.73**

[1]From residue 7 to residue 77

[2]Residues 13-28, 38-50 and 53-75

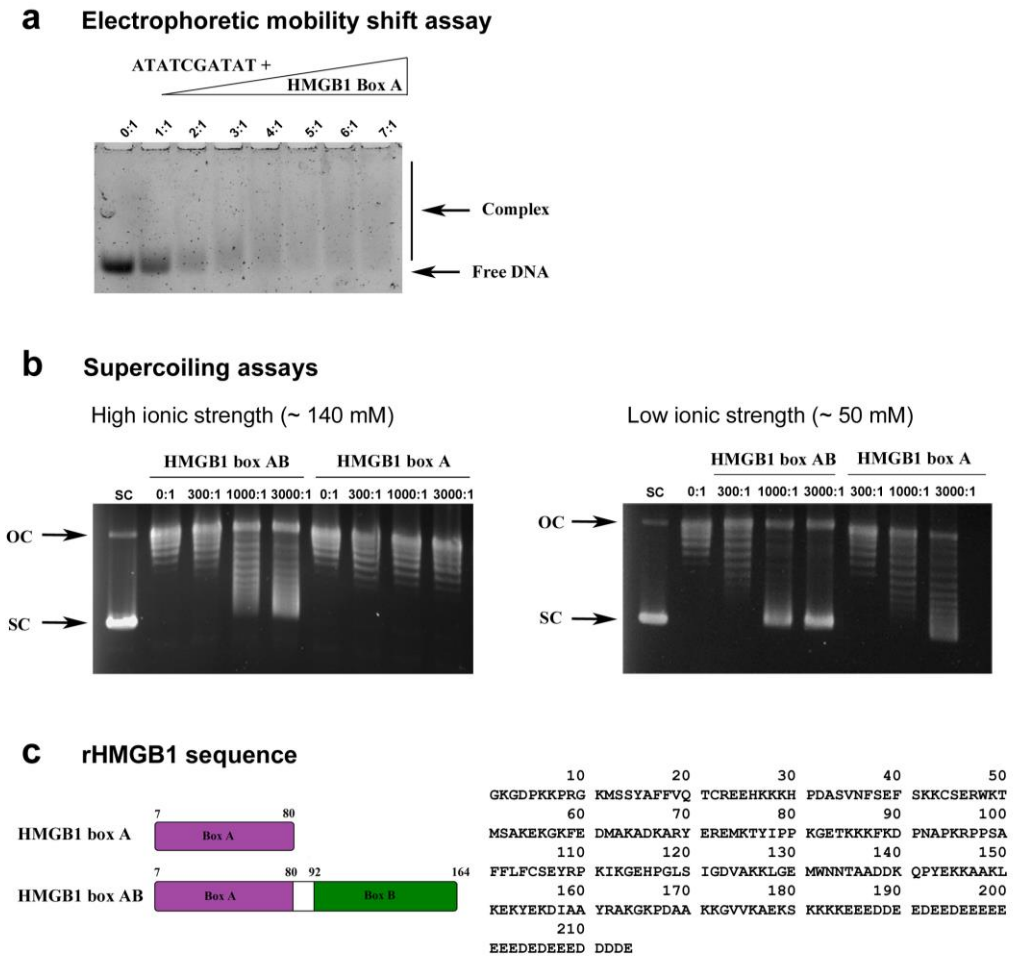

Figure S1 (a). EMSA showing box A binding to d(ATATCGATAT)$_2$. Protein:DNA molar ratios (μM:μM) are indicated. Interaction is evidenced by a retardation of the bands in the gel. Diffuse bands are commonly observed for non-sequence-specific proteins in EMSA, due to dissociation of the complexes during electrophoresis. (b). Supercoiling assays for the HMGB1 didomain box AB (HMGB1 lacking the acidic tail)* and box A at high and low ionic strength. Indicated with arrows are the supercoiled (SC) and the nicked form (open circular, OC) of the DNA. The introduction of negative supercoiling by box A is not observed at high ionic strength. However at low ionic strength it is clearly seen that box A at ~3 fold concentration introduces a degree of negative supercoiling similar to that introduced by the didomain box AB. The negative supercoiling introduced by these HMG-box domains is correlated with their unwinding activity. The negatively supercoiled topoisomers were confirmed by comparing the low ionic strength gel with the equivalent one in the presence of chloroquine (data not shown). (c) Schematic representation of box A and box AB fragments and rat HMGB1 full sequence. (*) HMGB1 didomain box AB (Lys7 to Lys164) was expressed and purified as described for box A, using an extinction coefficient of 20700 $M^{-1}cm^{-1}$.

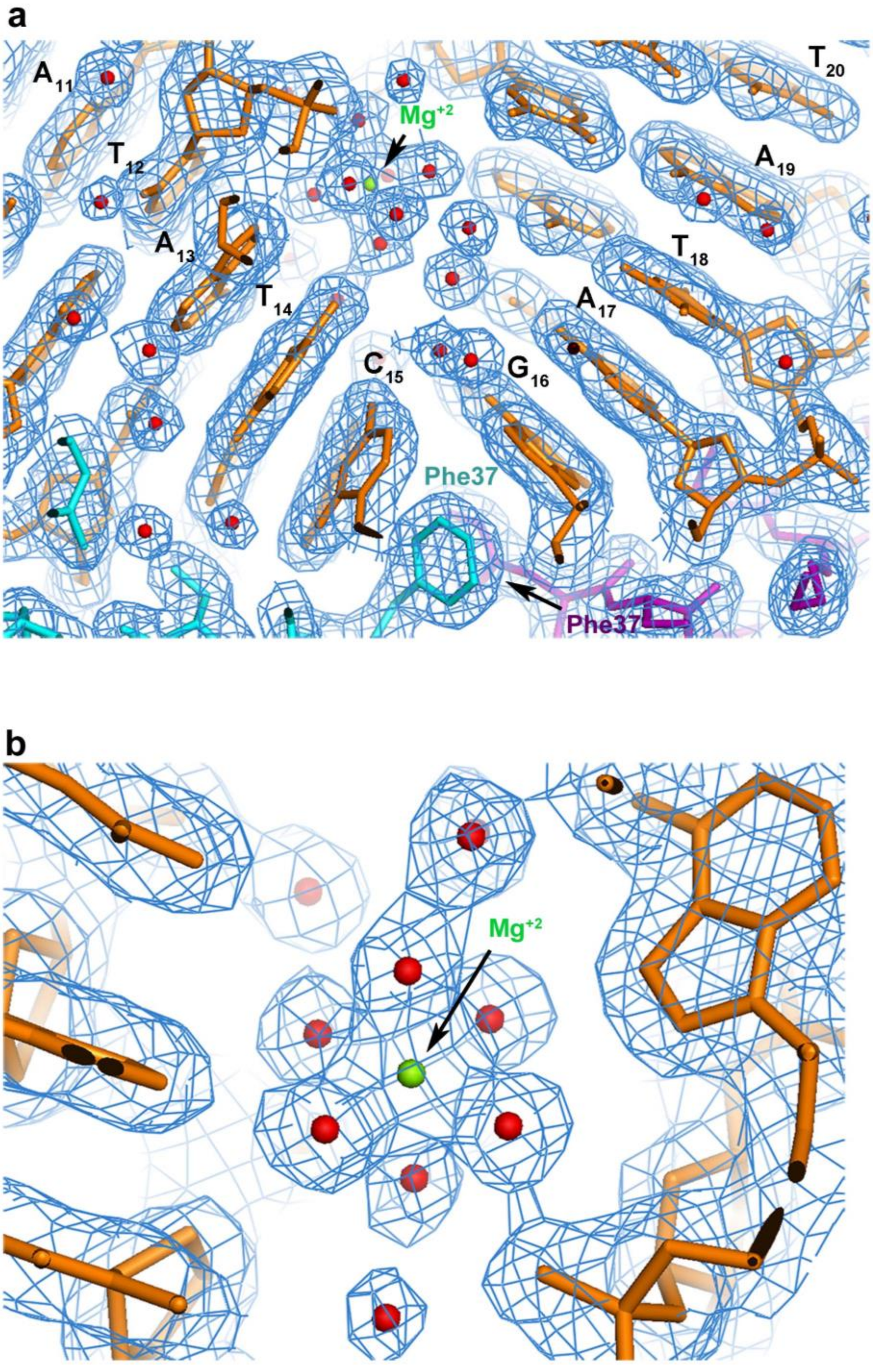

Figure S2 2Fo-Fc electron density maps at 1σ showing a magnesium ion interacting with the DNA. (a) Electron density map showing the central kink. (b) The Mg ion is located in the concave face that creates the bent DNA, making two water-mediated hydrogen bonds to the phosphate backbone.

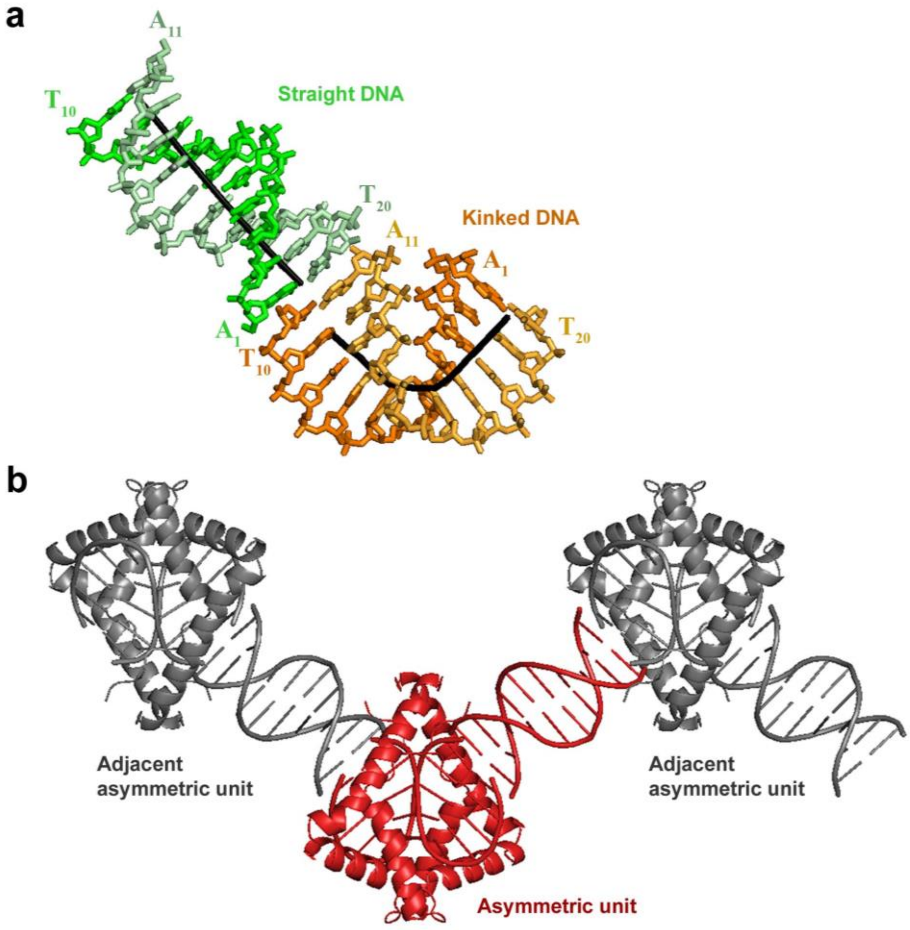

Figure S3 Structure of box A bound to the decamer ATATCGATAT. (a) DNA bending. One straight (green) and one kinked duplex (orange) with their axis are represented (CURVES/Pymol). In the kinked DNA, the $C_5G_6/C_{15}G_{16}$ base step has a roll angle of 74.85º, twist angle of 4.82º and rise of 6.64 Å, compared to 0.60º, 36.00º and 3.32 Å standard values for B-DNA, respectively. Also, C3'-*endo* sugar puckers, characteristic of A-DNA are found in nucleotides A_3, T_4, C_5, A_{13}, T_{14} and C_{15}. (b) Overall view of the asymmetric unit within the packing environment of the crystal. The two DNA decamers and the two box A domains in the asymmetric unit are shown in red, and the adjacent symmetry mates are shown in grey. The two box A domains bind and bend by approximately 85º the kinked DNA, whereas the straight DNA remains almost unbound (only makes two hydrogen bonds with Lys7 and Tyr77).

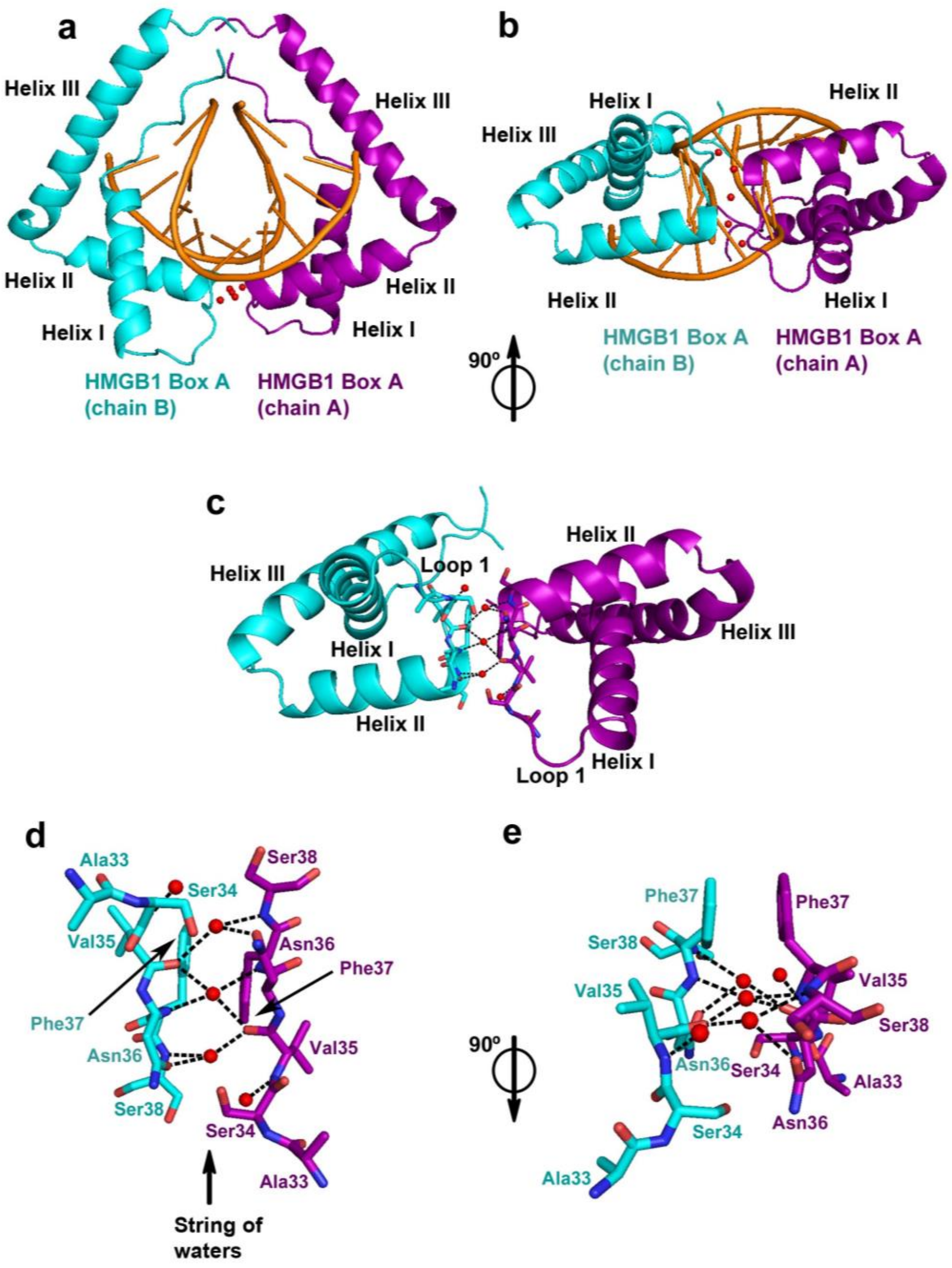

Figure S4 The dimerization interface contains a string of water molecules between loop 1 of both box A domains. **(a)** Side view of the complex box A-DNA showing the water molecules implicated in the water mediated hydrogen bonds between both domains. Proteins and DNA are colored as in Fig. 1 and waters are shown as red spheres. **(b)** Bottom view. **(c)** General view of the water mediated hydrogen bonding between domains. Residues implicated are drawn as sticks and hydrogen bonds are indicated as dashed lines. **(d)** Close up view of the water mediated interactions. The residues Ser34, Val35, Asn36, Phe37 and Ser38 of chain A (purple) and chain B (cyan) are involved in a water network interconnecting both domains. **(e)** Side view of **(d)**.
